# Wenzi Jiedu Recipe ameliorates colorectal cancer by remodeling the gut microbiota and tumor microenvironment

**DOI:** 10.3389/fonc.2022.915498

**Published:** 2022-09-23

**Authors:** Wenli Qiu, Tianqing Sang, Haibin Chen, Hongli Zhou, Zhongqiu Wang, Hongguang Zhou

**Affiliations:** ^1^ Department of Radiology, Affiliated Hospital of Nanjing University of Chinese Medicine, Nanjing, China; ^2^ The First Clinical Medical College, Henan University of Chinese Medicine, Zhengzhou, China; ^3^ Science and Technology Department, Jiangsu Collaborative Innovation Center of Traditional Chinese Medicine Prevention and Treatment of Tumor, Nanjing University of Chinese Medicine, Nanjing, China; ^4^ College of Pharmacy, Nanjing University of Chinese Medicine, Nanjing, China; ^5^ Department of Oncology, Affiliated Hospital of Nanjing University of Chinese Medicine, Nanjing, China

**Keywords:** traditional chinese medicine, wenzi jiedu recipe, colorectal cancer, gut microbiota, tumor microenvironment

## Abstract

**Introduction:**

Wenzi Jiedu Recipe (WJR), traditional Chinese medicine (TCM) formula, has been proven to be clinically useful in the treatment of colorectal cancer (CRC). However, its underlying mechanisms are still elusive, which limits its wider application. Thus, we aimed to evaluate the effect of WJR on CRC and elucidate mechanisms underlying its action.

**Methods:**

Network pharmacology was employed to clarify the “herb-active ingredient-target” network of WJR. The 16S rDNA sequencing method was used to analyze the changes of gut microbes mediated by WJR in tumor-bearing mice with CRC. The proportions of CD4+ T cell and CD8+ T cell were measured by flow cytometry. Levels of the cytokines interleukin (IL)-10, interferon (IFN)-γ, and tumor necrosis factor (TNF)-α were assessed by immunohistochemistry and enzyme-linked immunosorbent assay (ELISA).

**Results:**

WJR showed significant anti-CRC effects both *in vitro* and *in vivo*. Network pharmacology revealed that WJR exerts anti-CRC therapeutic effect on multiple targets and signaling pathways. Gut microbiota analysis revealed that WJR therapy significantly enriched for *Oscillibacter* and *Bacteroides_acidifacien*. In particular, we found that WJR significantly increased the proportion of CD8+ T cells and the expression of immune-associated cytokines IL-10, IFN-γ, and TNF-α.

**Conclusion:**

The regulation of gut microbiota by WJR may be the breakthrough point to clarify its mechanism of action in the treatment of CRC, and it has a good prospect of clinical application.

## Introduction

Colorectal cancer (CRC) is the third most common cancer worldwide and the third leading cause of cancer-related death, which affects over 200 000 people per year (1). In recent years, with the social development and changes in lifestyle, the incidence and mortality rates of CRC have increased rapidly, especially in developing countries (2). Despite advances in the treatment of CRC, the curative effect of CRC patients is unsatisfactory. It may be because CRC is caused by a combination of multi-factors, and monotherapies often fail to produce the desired efficacy (3). Traditional Chinese medicine (TCM) has been constructed for thousands of years in Asia, as a unique ancient medical science, and has been widely promoted in recent decades by promoting homeostasis to treat various diseases. Compared with monotherapies, TCM has the characteristics of “multi-components” and “multi-targets” and, thus, often exerts better curative effect on complex diseases (4).At present, increasing evidence that TCM could help alleviate CRC indicates that TCM has excellent potential in the treatment of CRC (5).

Composed of Astragali Preparata, Atractylodes, Coicis Semen, *Agrimonia pilosa*, Sparganii Rhizoma, Rhizoma Curcumae, Sophorae Flavescentis Radix, and Coptidis Rhizoma, Wenzi Jiedu Recipe (WJR) is a famous TCM prescription based on cancer toxin theory and yin and yang theory (6), combined with the Wenzi method and detoxification method. The Wenzi Jiedu method has been used to treat malignant tumors since ancient China. Although the efficacy of WJR in the treatment of CRC has been confirmed by many clinical appliances, its underlying mechanisms are still elusive, which limits its promotion.

As an integral part of the human body, the microbiota is mainly distributed in the oral cavity, skin, and gastrointestinal tract, which can regulate immunity and maintain intestinal physiological functions (7). Intestinal flora is equivalent to a virtual organ of the body, in which the colorectum is the organ with the highest distribution density of intestinal flora, and the imbalance of intestinal flora can induce colorectal disorder (8). In recent years, increasing studies have revealed that gut microbiota plays an important role in the occurrence and progression of CRC (9). Compared with normal people, the dominant microbiota in CRC patients is some pathogenic bacteria, such as *Bacteroides fragilis* and *Fusobacterium nucleatum* (10). Li Lv et al. found that the combination therapy with anti-mouse PD-1 mAb and Gegen Qinlian decoction (GQD) may ameliorate CRC *via* enriching beneficial bacteria, such as *s:uncultured_organism_g:norank_f:Bacteroidales_S24-7_group* and *s:Bacteroides_acidifaciens* (11). Meanwhile, TCM can play its role by regulating the composition of the intestinal flora (12). Sini Decoction (SND) can effectively intervene in the development of CRC by regulating the intestinal microbiota structure *via* enriching *Bacillus coagulans*, *Lactobacillus*, and *Bifidobacterium* and reducing *Bacteroides fragilis* and *Sulphate-reducing bacteria* (13).

Thus, taking the systems pharmacology platform and gut microbiota as the entry point, the purpose of the present work is to interpret the action mechanism of WJR in the treatment of CRC. We hope that the results of this research may not only improve the comprehension of CRC pathogenesis and the pharmacological basis of WJR but also provide theoretical support for promoting the application of TCM in the treatment of complex diseases.

## Materials and methods

### WJR preparation

WJR, containing Astragali Preparata (20g), Atractylodes (20g), Coicis Semen (30g), *Agrimonia pilosa* (30g), Sparganii Rhizoma (10g), Rhizoma Curcumae (10g), Sophorae Flavescentis Radix (5g), and Coptidis Rhizoma (3g), was purchased from Bozhou Traditional Chinese and Western Medicine Co., Ltd. (Anhui, China) and was identified by an experienced pharmacist. WJR was concentrated to 2.0 g/ml according to the standard method and stored at 4°C. Oxaliplatin was purchased from Shanghai Yuanye Biotechnology Co., Ltd (Shanghai, China).

### Preparation of medicated serum

24 adult Sprague-Dawley (SD) rats (220–250 g) were randomly divided into four groups and administered different doses of WJR: control group (normal saline), low-dose group (WJR 5.0 g/kg), middle-dose group (WJR 10.0 g/kg), and high-dose group (WJR 15.0 g/kg). Rats gavaged twice a day for 3 days. Within 1 h after the last gavage, blood samples were collected through retinal venous plexus and centrifuged to separate serum, and filtrated through a 0.22-μm filter. Medicated serum was stored at –80°C.

### Cell culture

Human colorectal carcinoma cell DLD-1 was obtained from the Cell Bank of the Chinese Academy of Sciences (Shanghai, China) and cultured in RPMI 1640 medium containing 10% fetal bovine serum, 100 μg/ml streptomycin, and 100 U/ml penicillin (all from Gibco, MA, USA) at 37°C with 5% CO_2_. Cells were incubated with a medium containing different concentrations of WJR-containing serum to explore the effect of WJR on DLD-1 cells.

### Cell proliferation and apoptosis assays

MTT assay was performed to determine the effect of WJR on cell proliferation. Briefly, DLD-1 cells treated with a medium containing different concentrations of drug serum were seeded in 96-well plates at a density of 1×10^4^/pore and incubated for 48 h. Then MTT (Sigma St. Louis, MO, USA) was added to wells and further incubated for 4 h. Subsequently, 150 µL DMSO (Sigma St. Louis, MO, USA) was added to each well. The optical density (OD) was detected with a microplate reader (Thermo Scientific, Waltham, MA, USA) at a wavelength of 492 nm.

Hoechst 33258 staining was implemented to detect apoptosis of DLD-1 cells. DLD-1 cells treated with a medium containing different concentrations of drug serum were seeded in six-well plates and incubated for 48 h. After treatment, the DLD-1 cells were fixed with 4% formaldehyde for 30 min. The fixed liquid discarded, the cells were stained with Hoechst 33258 (Beyotime Biotechnology, Shanghai, China) for 30 min. The cell morphology was observed and photographed under inverted fluorescence microscope (Olympus Co. Japan).

Flow cytometry assay was performed to detect the presence of apoptotic cells. DLD-1 cells treated with medium containing different concentrations of drug serum were seeded in six-well plates at a density of 1 × 10^6^/pore and incubated for 48 h. The obtained cells were resuspended in binding buffer and stained with Annexin V-FITC/PI (BD Pharmingen™, San Diego, CA, USA), and the signal was detected by flow cytometer (BD Biosciences).

Western blot assay was implemented to analyze the expression of apoptosis-related protein. DLD-1 cells were divided into four groups as aforementioned and the cell proteins were harvested with cell lysis solution. Protein concentration was quantified by the BCA protein assay according to the manufacturer’s instructions. Extracted proteins (50 µg) were loaded into the wells of SDS-PAGE gels and transferred onto the PVDF membrane. The membranes were incubated with primary antibodies (dilution 1:1,000; Bax cat.no.ab32503; bcl-2 cat.no.ab201335; Cell Signaling Technology, Cambridge, UK) overnight at 4°C and then incubated with horseradish peroxidase-labeled secondary antibody (dilution 1:5,000; cat.no.ab191866; Cell Signaling Technology, Cambridge, UK) at room temperature for 30 min. Signals were visualized by enhanced chemiluminescence (ECL) and analyzed using Image Lab software 4.1 (Bio-Rad, CA, USA). β-Actin was used as the internal reference for correction. All experiments were performed in triplicate.

### Animal study

The animal experimental protocol was approved by the Ethics Committee of the Affiliated Hospital of Nanjing University of Chinese Medicine (SYXK 2014–0001) and followed the guideline for laboratory animals in the Declaration of Helsinki. We purchased 30 BALB/c mice (male, 18–20 g, 4–6 weeks) from Changzhou Cavens Experimental Animal Co., Ltd., and fed them under specific pathogen-free conditions. To establish the xenograft tumor transplantation model, CT26 cells were suspended in an RPMI 1640 medium (1×10^6^ cells/mouse) and administered subcutaneously into the left axillary region of the mice. When the tumors reached 50 mm^3^ in size, the mice were randomly divided into five groups (six mice in each group): ① control group (NC, equal volume of physiologic saline); ② low-dose group (LD, orally gavaged with 5 g/kg body weight/day WJR); ③ middle-dose group (MD, orally gavaged with 10 g/kg body weight/day WJR); ④ high-dose group (HD, orally gavaged with 15 g/kg body weight/day WJR); ⑤ positive group (PC, administered with 8 mg/kg body weight/day oxaliplatin). The doses were administrated once a day for 14 days. The tumor volume and tumor growth inhibition rate (TGI) were calculated based on the formula of volume = length × width^2^ × 0.5 and TGI = (volume in control group – volume in treated group)/volume in control group ×100%.

### Histopathology

Tumor samples were harvested from each group and fixed with 4% paraformaldehyde, and then embedded in paraffin. The sections were stained with hematoxylin and eosin (H&E) and the morphologic changes of tumor sections were observed under an optical microscope.

### Potential anti-tumor mechanism of WJR for CRC

The corresponding targets and active ingredients of WJR were data-mined from TCMSP (http://lsp.nwu.edu.cn/) and TCMID (http://www.megabionet.org/tcmid/), and the candidate compounds were screened based on oral bioavailability (OB) ≥30%, drug-likeness (DL) ≥0.18, and half-life (HL)≥4. According to the systematic drug targeting approach by Yu et al., we obtained the corresponding targets for the candidate compounds in WJR.

We searched for the keywords ‘colorectal cancer’, ‘rectal cancer’, and ‘colon cancer’ in the GeneCards database (https://www.genecards.org/), OMIM database (http://www.omim.org/), and TTD database (https://db.idrblab.org/ttd/) to identify CRC-related targets.

Subsequently, the interaction network of compound-target and CRC-related targets were constructed and visualized using the Cytoscape 3.7.2 software. The protein–protein interaction (PPI) network was constructed using the BioGRID and then was further visualized using the Cytoscape. After merging the PPI network, the Analyze Network plug-in in Cytoscape was applied to extract and calculate the topological parameters of nodes in the interaction network such as degree centrality (DC), betweenness centrality (BC), and closeness centrality (CC) to determine the main active ingredients and targets of WJR in the treatment of CRC.

### GO and KEGG enrichment analysis

Gene Ontology (Go) enrichment analysis was mainly utilized to symbolize the functions of genes, and Kyoto Encyclopedia of Genes and Genomes (KEGG) enrichment analysis was used to obtain the signaling pathways enriched by the targets. GO and KEGG enrichment analysis were constructed using the Database for Annotation, Visualisation and Integrated Discovery (DAVID) to annotate the functions of the key targets. The Benjamini–Hochberg method was used to calculate and correct the p-value, and less than 0.01 was selected as the cutoff criterion.

### Fecal gut microbial analysis

Feces of the mice were collected after administrating with a corresponding dose of agents for gut microbiota analyses. Stool DNA was extracted using PowerSoil DNA Isolation Kit (MOBIO, Carlsbad, CA) according to the manufacturer’s instructions. The 16S rDNA V3–V4 region was amplified by PCR using the 343F and 798R primers (343F: TACGGRAGGCAGCAG; 798R: AGGGTATCTAATCCT); then we performed the quantification, qualification, and purification of the PCR product. Sequencing was performed on the Illumina MiSeq (Illumina Inc., San Diego, CA) based on the previous study. FLASH (Version 1.2.11) was used for quality filtering of the 16S rRNA sequencing data. Operational taxonomic units (OTUs) were picked at 97% sequence similarity, and then the taxonomical identification was aligned using the Silva database (Release 132). Moreover, the RDP classifier was applied to assign 16S rRNA sequences at a given taxonomic rank.

### Flow cytometry

ALL peripheral blood was collected from the orbital vein plexus, stained with ANTI-CD3-FITC, ANTI-CD4-PE, and ANTI-CD8-PE monoclonal antibodies (BD Biosciences, San Jose, CA), and incubated in the dark at room temperature for 15 min. Then, erythrocytes were lysed with FACS lysing buffer (BD Biosciences, San Jose, CA) incubated in the dark for 10 min. The mixture was measured with a Fortessa Flow Cytometer (BD Biosciences, San Jose, CA) and the data were analyzed with the FlowJo v10 software (Tree Star Inc., Ashland, OR).

### Immunohistochemistry and enzyme-linked immunosorbent assay (ELISA)

Xenograft tumors were harvested from the mice, fixed in 4% paraformaldehyde, embedded into paraffin, and then cut into 4-μm-thick sections. Sections were stained with hematoxylin. The slides were blocked with goat serum at 37°C for 20 min and incubated with the primary antibody (1:100, Cell Signaling Technology, Cambridge, UK) at 4°C overnight. Then, each sample was incubated with the secondary antibody (Cell Signaling Technology, Cambridge, UK) for 30 min at room temperature, followed by staining with a DAB substrate kit (Agilent Technologies, Santa Clara, CA). After dehydrating and drying, the stained samples were observed under a microscope. The signals were analyzed with the ImageJ software.

The expression levels of cytokines including IL-10, IFN-γ, and TNF-α in mice serum were detected using ELISA kits (Sino Biological, Beijing, China). All the procedures of ELISA assays followed the manufacturer’s protocol.

### Statistics analysis

Statistical analyses were performed using the SPSS 20.0 software. Quantitative data were presented as mean ± standard deviation (SD) from at least three independent experiments. Comparisons between two groups were performed by two-tailed unpaired *t*-test. Multiple comparisons were performed by one-way analysis of variance (ANOVA) with *post hoc* contrasts by the Student-Newman-Keuls test. A level of p < 0.05 was considered statistically significant.

## Results

### Cell proliferation and apoptosis assays

**Figure 1:**
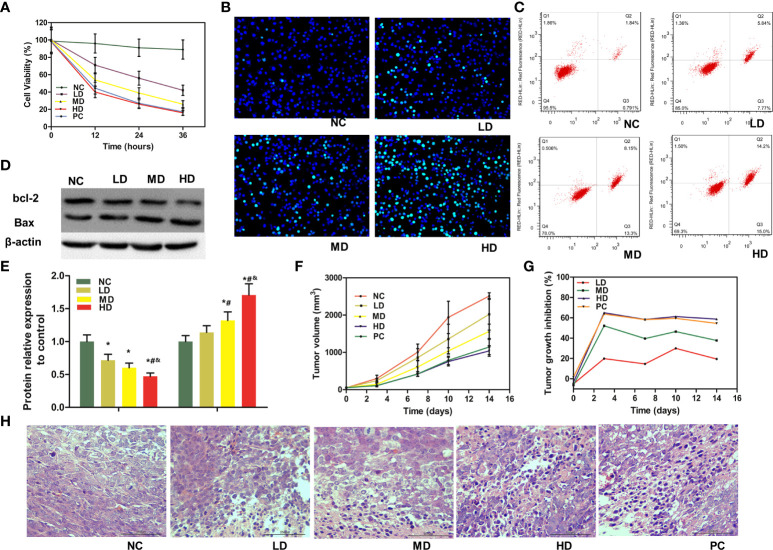
Anti-tumor efficacy of WJR *in vitro* and *in vivo*. **(A)** Cell viability was determined by MTT assay in DLD-1 cells under WJR. **(B)** Hoechst 33258 staining and **(C)** flow cytometry assay were used to detect apoptosis of DLD-1 cells. **(D, E)** Western blot assay was implemented to analyze the expression of apoptosis-related protein Bax and bcl-2. **(F)** Tumor volume changes in mice treated with different groups. **(G)** TGI of mice in different groups. **(H)** H&E of tumor samples, and areas of hyperchromatic nuclei and infiltration of inflammatory cells represent necrosis. NC, control group; LD, low-dose group of WJR; MD, middle-dose group of WJR; HD, high-dose group of WJR; PC, positive group; WJR, Wenzi Jiedu Recipe. H&E, hematoxylin and eosin; TGI, tumor growth inhibition rate. ^*^vs. NC, *p* < 0.05; ^#^vs. LD, *p* < 0.05; ^&^vs. MD, *p* < 0.05.

MTT assay showed that WJR obviously suppressed the proliferation ability of DLD-1 cells in a dose-dependent manner compared to the NC group (**Figure 1A**). Hoechst 33258 staining revealed that an increasing proportion of apoptotic cells was found following WJR treatment in a dose-dependent manner (**Figure 1B**). Flow cytometry was employed to determine the apoptosis rate of cells. As shown in **Figure 1C**, compared to the NC group, the percentage of apoptotic cells was significantly increased following WJR treatment, and the inhibitory effect of the high dose of WJR was the best. In addition, the expression of pro-apoptotic Bax protein was increased in a concentration-dependent manner following XJR treatment, accompanied by a decreasing expression of anti-apoptotic Bcl-2 protein (**Figures 1D, E**). Taken together, these results indicated that WJR has a significant anti-tumor effect *in vitro*.

### Anti-tumor efficacy of WJR *in vivo*

We investigated the *in vivo* anti-tumor efficacy of WJR with CT26 colorectal tumor xenograft models. As shown in **Figure 1F**, the tumors in the NC group grew rapidly. Compared with the NC group, the LD and MD group inhibited tumor progression to a certain extent. The HD and PC therapy had the strongest inhibitory effect on tumor progression. The tumor volume in the HD and PC groups were much smaller than that in the NC group, and there was no significant difference between the HD and PC groups. At the same time, we also performed the TGI score during treatment phase. We found that the TGI of the LD and MD groups were significant (**Figure 1G**). However, the TGI of the HD and PC groups were more significant, which was consistent with the results of the tumor volume.

To better demonstrate the *in vivo* anti-tumor efficacy of WJR on CRC, H&E staining was performed to investigate the histopathology changes of tumor samples. According to the pathological observation, the structure of tumor tissues in HD and PC groups suffered more apparent extensive damage, such as severe necrosis and intercellular spaces being widened irregularly; however, tumor tissues in the LD and MD groups had moderate damage (**Figure 1H**). Altogether, these results indicated that WJR could inhibit tumor progression of CRC *in vivo*, and high dose had stronger inhibitory effect.

### Potential anti-tumor mechanisms of WJR for CRC

**Figure 2:**
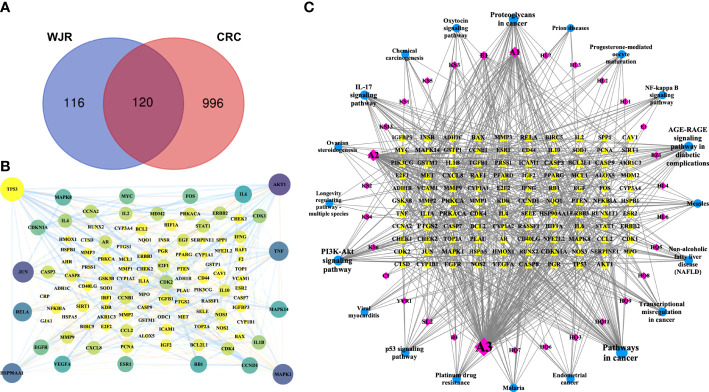
Potential anti-tumor mechanisms of WJR for CRC. **(A)** Venn diagram of WJR-related targets intersected with 1116 CRC-related targets. **(B)** PPI network of candidate WJR targets for CRC treatment. **(C)** PPI network of WJR-active ingredients, CRC-related targets, and signaling pathways. Yellow, CRC-related targets; purple, WJR-active ingredients; blue, signaling pathways. WJR, Wenzi Jiedu Recipe; CRC, colorectal cancer.

TCMSP and TCMID databases were used to search for the chemical composition the eight herbs that constitute WJR. In total, WJR includes 493 chemicals. Based on the OB ≥30%, DL ≥0.18, and HL ≥4 screen criteria, we obtained 91 potential active compounds from WJR, of which 16 were from Astragali Preparata, 5 were from Atractylodes, 5 were from Sparganii Rhizoma, 3 were from Rhizoma Curcumae, 3 were from *Agrimonia pilosa*, 10 were from Coptidis Rhizoma, 42 were from Sophorae Flavescentis Radix, and 4 were from Coicis Semen (**Table S1**). Corresponding relationships between therapeutic targets and active compounds, deleting duplicate targets, 81 active compounds, and 236 potential therapeutic targets were obtained (**Table S2**). We next identified CRC-related targets from GeneCards, OMIM, and TTD databases. Based on the median values for Score, we obtained 1116 CRC-related targets (**Table S2**). After intersecting the 236 WJR-related targets with 1116 CRC-related targets, 120 mutual targets were identified as crucial anti-CRC WJR targets (**Figure 2A**), and the PPI network was constructed (**Figure 2B**).

**Figure 3:**
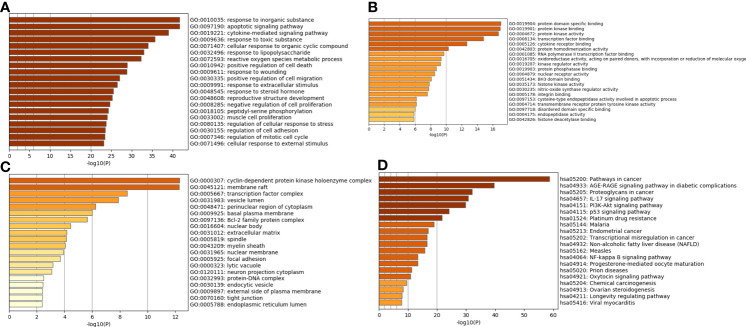
GO and KEGG enrichment analysis of the potential WJR targets for CRC treatment. GO enrichment analysis of the top 20 terms of **(A)** BP, **(B)** MF, and **(C)** CC. **(D)** The top 20 pathways of the KEGG enrichment analysis. BP, biological process; MF, molecular function; CC, cellular component. WJR, Wenzi Jiedu Recipe; CRC, colorectal cancer; BP, biological process; MF, molecular function; CC, cellular components.

DAVID was used for functional annotation of the crucial target genes. The GO enrichment analysis included three parts: biological process (BP), molecular function (MF), and cellular components (CC). The top 20 terms of BP, MF, and CC are shown in **Figures 3A–C**. The GO enrichment analysis showed that the crucial target genes mainly involved biological processes such as response to inorganic substance, cytokine-mediated signaling pathway, response to toxic substance, and cellular response to organic cyclic compound; molecular function such as protein domain specific binding, protein kinase binding, protein kinase activity, transcription factor binding, and cytokine receptor binding; and cellular components such as cyclin-dependent protein kinase holoenzyme complex, membrane raft, transcription factor complex, vesicle lumen, and perinuclear region of cytoplasm. The top 20 pathways of the KEGG enrichment analysis are shown in **Figure 3D**, such as pathways in cancer, AGE-RAGE signaling pathway in diabetic complications, proteoglycans in cancer, IL-17 signaling pathway, and PI3K-Akt signaling pathway. Therefore, we postulated that WJR exerts an anti-CRC therapeutic effect on multiple targets and signaling pathways through its complex active ingredients.

We then constructed a PPI network of WJR-active ingredients, CRC-related targets, and signaling pathways with CytoScape (**Figure 2C**). After merging the PPI network, we extracted and calculated the topological parameters of nodes in the interaction network. As shown in **Table 1**, six active ingredients (quercetin, luteolin, kaempferol, formononetin, 8-Isopentenyl-kaempferol, and beta-sitosterol) were selected with high node scores, suggesting that these compounds may play a major role in the anti-CRC effect of WJR.

**Table 1:** Topological parameters of main active ingredients of WJR.

MOL ID	Molecule Name	DC	BC	CC
MOL000098	Quercetin	135	0.20782338	0.43526171
MOL000006	Luteolin	59	0.08378578	0.40409207
MOL000422	Kaempferol	46	0.11219171	0.41688654
MOL000392	Formononetin	21	0.04652085	0.35505618
MOL003542	8-Isopentenyl-kaempferol	11	0.02185093	0.34573304
MOL000358	Beta-sitosterol	11	0.0194422	0.36155606

### WJR modulated the gut microbiome composition

**Figure 4:**
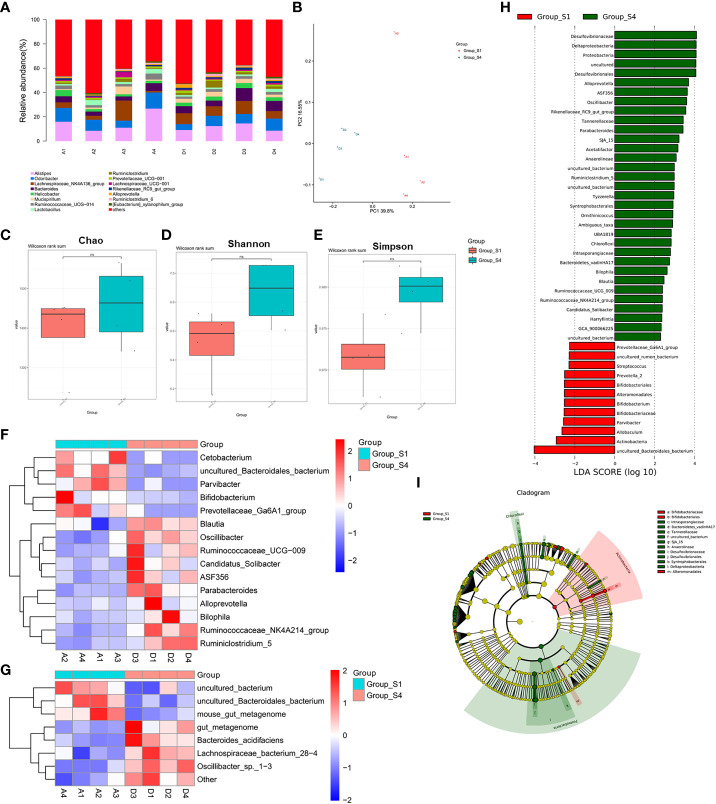
WJR modulated the gut microbiome composition. **(A)** Bar plot of the phylogenetic composition of gut microbiome at the genus level. **(B)** PCoA in different groups (A1-4, S1 group; D1-4, S4 group). Alpha diversity of the gut microbiome between the control group and the high dose of the WJR group based on **(C)** Chao, **(D)** Shannon and **(E)** Simpson indices. **(F)** Wilcoxon rank-sum test was performed for bacterial taxa at the genus level **(G)** and species level. **(H)** LEfSe analysis. **(I)** Taxonomic cladogram from LEfSe. S1, control group; S4, high-dose of WJR group; WJR, Wenzi Jiedu Recipe. ns, not significant.

Based on the results of anti-tumor efficacy of WJR, we prospectively collected the gut microbiome samples from the control group (NC, n = 4) and the high-dose WJR group (HD, n = 4). Firstly, we evaluated the composition of the gut microbiome in all samples, noting the relative diversity of communities in the different treatment groups (**Figure 4A**). Principal component analysis (PCoA) showed that the microbial community structure of the HD group and NC group was clearly separated, indicating that the core microbiota had significant changes after WJR treatment (**Figure 4B**). We found that there was no significant difference in the alpha diversity of the gut microbiome between the HD group and the NC group based on Chao, Shannon, and Simpson indices (**Figures 4C–E**). We then sought to explore if differences existed in the gut microbiomes after WJR treatment in CRC. Wilcoxon rank-sum test was performed for bacterial taxa at the genus level; we found that *Cetobacterium*, *uncultured_Bacteroidales_bacterium*, *Parvibacter*, *Bifidobacterium*, and *Prevotellaceae_Ga6A1_group* were enriched in the NC group, whereas *Blautia*, *Oscillibacter*, *Ruminococcaceae_UCG−009*, *Candidatus_Solibacter*, *ASF356*, *Parabacteroides*, *Alloprevotella*, *Bilophila*, *Ruminococcaceae_NK4A214_group*, and *Ruminiclostridium_5* were enriched in the HD group (**Figure 4F**). At the species level, we observed that the NC group was enriched in *uncultured_bacterium*, *uncultured_Bacteroidales_bacterium*, and *mouse_gut_metagenome*, whereas the HD group was enriched in *gut_metagenome*, *Bacteroides_acidifaciens*, *Lachnospiraceae_bacterium_28−4*, and *Oscillibacter_sp._1−3* (**Figure 4G**). To further explore these findings, the high-dimensional class comparison was performed *via* linear discriminant analysis effect size (LEfSe). We found differentially abundant bacteria in the gut microbiomes between the different treatment groups, with *uncultured_Bacteroidales_bacterium*, *Actinobacteria*, and *Allobaculum* enriched in the NC group and *Desulfovibrionaceae*, *Deltaproteobacteria*, *Proteobacteria*, and *Desulfovibrionales* enriched in the HD group (**Figures 4H, I**).

### WJR treatment enhanced anti-tumor immunity

**Figure 5:**
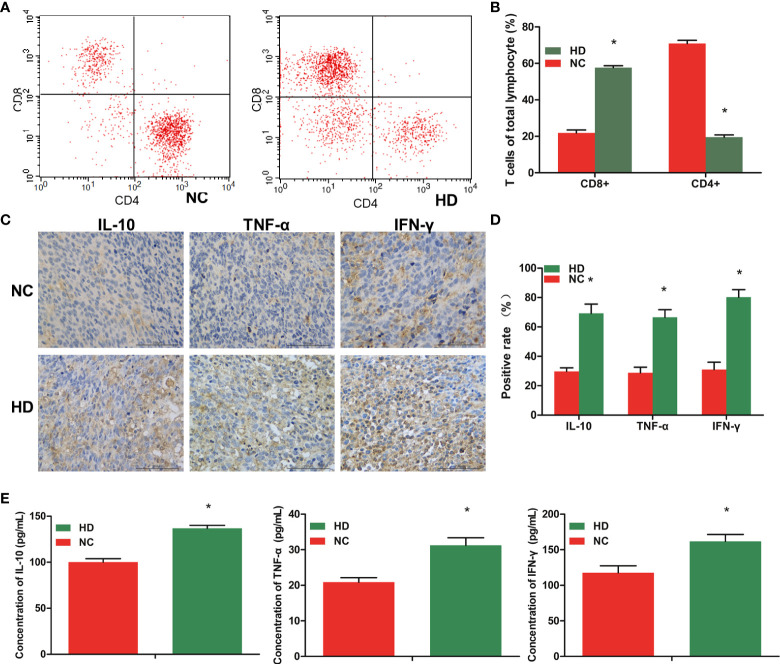
WJR treatment enhances anti-tumor immunity. **(A, B)** Flow cytometry was used to analyze the proportion of CD8^+^ T-cell and CD4+T cell in the total peripheral blood lymphocyte cells. **(C, D)** The expression levels of IL-10, IFN-γ, and TNF-α in the tumor tissue was analyzed by immunohistochemistry. **(E)** The expression levels of IL-10, IFN-γ, and TNF-α in serum was analyzed by ELISA. (n = 4). WJR, Wenzi Jiedu Recipe; NC, control group; HD, high-dose group of WJR. ^*^vs. NC, *p* < 0.05.

Peripheral blood lymphocyte cells were collected and stained, and the systemic immune response was analyzed by flow cytometry. The proportion of CD8^+^ T-cell in the total peripheral blood lymphocyte cells was significantly higher in the HD group than in the NC group, and the CD4+T cell proportion in the HD group tended to be lower than that in the NC group (**Figures 5A, B**).

To explore whether tumor microenvironment in mice is changed after WJR treatment, we further evaluated the tumor-associated immune by measuring the levels of several indicator immune-associated cytokines. The immunohistochemistry results revealed that the mice after WJR treatment had higher densities of IL-10, IFN-γ, and TNF-α than the NC group in the tumor tissue (**Figures 5C, D**). The ELISA results also revealed that the mice after WJR treatment had higher levels of IL-10, IFN-γ, and TNF-α in serum, consistent with the above results (**Figure 5E**). These results suggested that WJR therapy could effectively blockade in CRC.

## Discussion

TCM is a precious heritage of mankind which has been constructed for more than 3000 years in Asia. Herbal medicine has gradually become an alternative medicine accepted worldwide and is widely used in the treatment of complicated diseases (14). As one of the acclaimed TCM preparations, WJR is composed of Astragali Preparata, Atractylodes, Coicis Semen, *Agrimonia pilosa*, Sparganii Rhizoma, Rhizoma Curcumae, Sophorae Flavescentis Radix, and Coptidis Rhizoma. Although it has been clinically proven that WJR can prolong the survival of patients with CRC and improve the quality of life of patients, its mechanism has not yet been clarified. In the present study, we analyzed the anti-tumor efficacy of WJR *in vitro* and *in vivo* and explored its possible mechanism of action in the treatment of CRC through network pharmacology, gut microbiome, and system immune response. Our results showed that WJR can effectively intervene in the progression of CRC by regulating the composition of the intestinal flora and restoring the system immune response.

WJR is a complex chemical system, characterized as “multi-component”, “multi-target”, and “multi-pathway”. Network pharmacology is a method to analyze the relationship between drugs, targets, and diseases, through the network method, to determine the mode of action of drugs (15). In the present study, a systems pharmacology approach was employed to clarify the “herb-active ingredient-target” network of WJR. We identified 120 significant targets as crucial anti-CRC WJR targets. Based on KEGG pathway enrichment, we found that the pathways in cancer, AGE-RAGE signaling pathway in diabetic complications, proteoglycans in cancer, IL-17 signaling pathway, and PI3K-Akt signaling pathway were the top 20 significant pathways. We also found that quercetin, luteolin, kaempferol, formononetin, 8-Isopentenyl-kaempferol, and beta-sitosterol had a high correlation in the network and may play a major role in the anti-CRC effect of WJR. Previous studies have shown that quercetin could inhibit CRC by inhibition metastasis, angiogenesis, cell cycle or regulation of signaling pathways (16). Atashpour et al. found that quercetin could induce cell cycle arrest and the apoptosis of CRC cells and enhance the anti-cancer effect of doxorubicin (17). Luteolin could suppress CRC cell metastasis by modulating the miR-384/pleiotrophin axis (18). Kaempferol had strong anti-oxidant, apoptotic, and cytotoxic effects against CRC HCT-116 cells (19), and could be used as a potential chemotherapeutic agent to overcome CRC drug resistance (20). Network pharmacological approach showed that formononetin was involved in regulating cancer-related metabolic pathways and suppressing proliferation to inhibit CRC (21). Beta-sitosterol is a phytosterol, which could mediate the p53/NF-κB/BCRP signaling pathway to alleviate multidrug resistance in the treatment of CRC and, combined with oxaliplatin, could be a potential way to improve CRC therapy efficacy (22). Therefore, we speculated that WJR exerts excellent anti-CRC effect on multiple targets and signaling pathways through its crucial active ingredients.

With the development of molecular ecology and high-throughput sequencing technology, increasing researchers have begun to concern about the function of the tumor microenvironment in CRC occurrence and development. The imbalance of gut micro-ecological has become a hot topic in the pathogenesis of CRC, which may provide an effective treatment of CRC. Thus, we then explored gut microbiota modulation by WJR in tumor-bearing mice. The 16S rDNA sequencing method was used to analyze the difference of gut microbiota in tumor-bearing mice with CRC before and after high-dose WJR treatment. Compared with pre-treatment, the abundance of *Oscillibacter*, *Bacteroides_acidifacien* was increased, while the abundance of *uncultured_Bacteroidales_bacterium* was decreased post-treatment. As beneficial bacteria, *Oscillibacter* could reduce intestinal inflammation by regulating immune cells (23). The content of *Bacteroidetes* in CRC patients was higher than that in normal people, which could be considered as a non-invasive marker for the early detection of CRC (24). *Bacteroides acidifacien* is the predominant bacteria that promote the production of IgA in the large intestine (25). In our study, *Bacteroides acidifaciens* was significantly enriched in the post-treatment group. Hence, we considered that *Bacteroides acidifaciens* is the intestinal “beneficial bacteria”, which may enhance host immunity. However, *uncultured_Bacteroidales_bacterium* was enriched in the control group, suggesting that it is probably “harmful bacteria”. A study in mice reported that *s:uncultured_Bacteroidales_bacterium_g:norank_f:Bacteroidales_S24-7_group* is a major bacterial group in the CRC group, which may be “unfavorable” (11). We demonstrated that WJR can increase beneficial bacteria and reduce harmful bacteria, which is consistent with previous studies that TCM may inhibit CRC by regulating gut microbiota (8).

Many researchers have discovered that the effect of intestinal flora is not confined to the intestine but also has a crucial regulatory effect on immune function (26). The abundance of *Fusobacterium nucleatum* in patients with CRC was found to be inversely proportional to the density of CD3+ T cells. It was reported that the abundance of *Megamonas* is closely associated to inflammatory response and immune function (27). These results indicated that the intestinal flora may be engaged in the regulation of immune response. In addition, most CRCs are not responsive to conventional immunotherapy. In our study, flow cytometry analysis showed higher CD8+ T-cells and lower CD4+ T-cells in the WJR post-treatment group than in the NC group. The proportion of CD8+ T-cells increased after WJR treatment, suggesting that WJR can play a role in tumor cytotoxicity by mobilizing the cellular immune system. IFN-γ is a pleiotropic cytokine, mainly produced by Th1 cells, NK cells, and cytotoxic T cells, which is involved in tumor clearance, dormancy, escape, and progression (28). TNF-α is also a multi-functional cytokine, which plays an important role in immune regulation and inflammatory response, and can participate in the regulation of tumor cell apoptosis, proliferation, angiogenesis, differentiation, and migration (29, 30). It is currently a cytokine with the strongest direct killing effect on tumor cells. Mice receiving WJR treatment had higher levels of IFN-γ and TNF-α in tumor tissues and in serum, suggesting enhanced host cellular immunity. IL-10 is a pleiotropic cytokine with anti-inflammatory property and immunoregulatory function, which is associated with proliferation and apoptosis of various cancers (31). The high level of IL-10 is an important indicator of the recovery of anergic T cell function (32). In this study, we speculated that WJR has the effect of protecting intestinal mucosal tissue and inhibiting inflammation; therefore, the level of IL-10 is increased after treatment, and the increased IL-10 expression suggests the restoration of T cell function and enhancement of host immunity. Combined with these changes, we considered that WJR may restrain CRC development through immune regulation.

In this study, WJR has shown significant anti-tumor effects both *in vivo* and *in vitro*, and no obvious side-effects were observed, indicating that WJR is a promising treatment option for CRC. We also found that WJR can regulate the structure and relative abundance of intestinal flora in tumor-bearing mice. Moreover, WJR could increase the proportion of CD8+ T cells and the expression of immune-associated cytokines IL-10, IFN-γ, and TNF-α. However, we currently have no evidence that there is a direct causal relationship between the ability of WJR to modulate the gut microbiota and its ability to regulate immune function and alleviate CRC, which will be the priority of our next research. We speculated that the regulation of gut microbiota by WJR may be the breakthrough point to clarify its mechanism of action in the treatment of CRC, and it has a good prospect of clinical application.

## Data availability statement

The data presented in the study are deposited in the NCBI repository, accession number PRJNA788167.

## Ethics statement

The animal study was reviewed and approved by Affiliated Hospital of Nanjing University of Chinese Medicine.

## Author contributions

Design of experiment: HgZ; paper drafting and *in vitro* experiment: WQ; establishment of mouse model and *in vivo* experiment: HC; data analysis: TS; paper correction: WQ, HlZ, and ZW. All authors read and approved the final manuscript.

## Funding

This work was supported by National Natural Science Foundation of China (No. 81973737, 82001883, and 81771899).

## Conflict of interest

The authors declare that the research was conducted in the absence of any commercial or financial relationships that could be construed as a potential conflict of interest.

## Publisher’s note

All claims expressed in this article are solely those of the authors and do not necessarily represent those of their affiliated organizations, or those of the publisher, the editors and the reviewers. Any product that may be evaluated in this article, or claim that may be made by its manufacturer, is not guaranteed or endorsed by the publisher.
