# Supplementary material for: Wenzi Jiedu Recipe ameliorates colorectal cancer by remodeling the gut microbiota and tumor microenvironment
Source: Front Oncol. 2022 Sep 23;12:915498. doi: 10.3389/fonc.2022.915498 (PMC9541612; doi:10.3389/fonc.2022.915498)
Supplement: Supplementary file 1 [file DataSheet_1.docx]

Supplementary Material

**Tables**

**Supplemental Table S1**. Potential active compounds from WJR

| **Herbname** | **MOL ID** | **Molecule Name** | **OB (%)** | **DL** | **HL** |
| --- | --- | --- | --- | --- | --- |
| Astragali Preparata | MOL000442 | 1,7-Dihydroxy-3,9-dimethoxy pterocarpene | 39.05 | 0.48 | 7.95 |
| Astragali Preparata | MOL000433 | \| [FA](http://tcmspw.com/molecule.php?qn=433) \| \| --- \| | 68.96 | 0.71 | 24.81 |
| Astragali Preparata | MOL000422 | [kaempferol](http://tcmspw.com/molecule.php?qn=422) | 41.88 | 0.24 | 14.74 |
| Astragali Preparata | MOL000417 | [Calycosin](http://tcmspw.com/molecule.php?qn=417) | 47.75 | 0.24 | 17.1 |
| Astragali Preparata | MOL000398 | [isoflavanone](http://tcmspw.com/molecule.php?qn=398) | 109.99 | 0.3 | 15.51 |
| Astragali Preparata | MOL000392 | [formononetin](http://tcmspw.com/molecule.php?qn=392) | 69.67 | 0.21 | 17.04 |
| Astragali Preparata | MOL000387 | [Bifendate](http://tcmspw.com/molecule.php?qn=387) | 31.1 | 0.67 | 17.96 |
| Astragali Preparata | MOL000380 | [(6aR,11aR)-9,10-dimethoxy-6a,11a-dihydro-6H-benzofurano[3,2-c]chromen-3-ol](http://tcmspw.com/molecule.php?qn=380) | 64.26 | 0.42 | 8.49 |
| Astragali Preparata | MOL000379 | [9,10-dimethoxypterocarpan-3-O-β-D-glucoside](http://tcmspw.com/molecule.php?qn=379) | 36.74 | 0.92 | 13.06 |
| Astragali Preparata | MOL000371 | [3,9-di-O-methylnissolin](http://tcmspw.com/molecule.php?qn=371) | 53.74 | 0.48 | 9 |
| Astragali Preparata | MOL000354 | [isorhamnetin](http://tcmspw.com/molecule.php?qn=354) | 49.6 | 0.31 | 14.34 |
| Astragali Preparata | MOL000296 | [hederagenin](http://tcmspw.com/molecule.php?qn=296) | 36.91 | 0.75 | 5.35 |
| Astragali Preparata | MOL000239 | [Jaranol](http://tcmspw.com/molecule.php?qn=239) | 50.83 | 0.29 | 15.5 |
| Astragali Preparata | MOL000211 | [Mairin](http://tcmspw.com/molecule.php?qn=211) | 55.38 | 0.78 | 8.87 |
| Astragali Preparata | MOL000098 | [quercetin](http://tcmspw.com/molecule.php?qn=98) | 46.43 | 0.28 | 14.4 |
| Astragali Preparata | MOL000033 | [(3S,8S,9S,10R,13R,14S,17R)-10,13-dimethyl-17-[(2R,5S)-5-propan-2-yloctan-2-yl]-2,3,4,7,8,9,11,12,14,15,16,17-dodecahydro-1H-cyclopenta[a]phenanthren-3-ol](http://tcmspw.com/molecule.php?qn=33) | 36.23 | 0.78 | 5.22 |
| Atractylodes | MOL000020 | [12-senecioyl-2E,8E,10E-atractylentriol](http://tcmspw.com/molecule.php?qn=20) | 62.4 | 0.22 | 6.07 |
| Atractylodes | MOL000021 | [14-acetyl-12-senecioyl-2E,8E,10E-atractylentriol](http://tcmspw.com/molecule.php?qn=21) | 60.31 | 0.31 | 5.32 |
| Atractylodes | MOL000022 | [14-acetyl-12-senecioyl-2E,8Z,10E-atractylentriol](http://tcmspw.com/molecule.php?qn=22) | 63.37 | 0.3 | 6.43 |
| Atractylodes | MOL000033 | (3S,8S,9S,10R,13R,14S,17R)-10,13-dimethyl-17-[(2R,5S)-5-propan-2-yloctan-2-yl]-2,3,4,7,8,9,11,12,14,15,16,17-dodecahydro-1H-cyclop[enta[a]phenanthren-3-ol](http://tcmspw.com/molecule.php?qn=33) | 36.23 | 0.78 | 5.22 |
| Atractylodes | MOL000072 | [8β-ethoxy atractylenolide Ⅲ](http://tcmspw.com/molecule.php?qn=72) | 35.95 | 0.21 | 8.34 |
| Sparganii Rhizoma | MOL001297 | [trans-gondoic acid](http://tcmspw.com/molecule.php?qn=1297) | 30.7 | 0.2 | 5.25 |
| Sparganii Rhizoma | MOL000449 | [Stigmasterol](http://tcmspw.com/molecule.php?qn=449) | 43.83 | 0.76 | 5.57 |
| Sparganii Rhizoma | MOL000392 | [formononetin](http://tcmspw.com/molecule.php?qn=392) | 69.67 | 0.21 | 17.04 |
| Sparganii Rhizoma | MOL000358 | [beta-sitosterol](http://tcmspw.com/molecule.php?qn=358) | 36.91 | 0.75 | 5.36 |
| Sparganii Rhizoma | MOL000296 | [hederagenin](http://tcmspw.com/molecule.php?qn=296) | 36.91 | 0.75 | 5.35 |
| [Rhizoma Curcumae](https://www.so.com/link?m=bxhhJwRTeWC/prtMCOClH8hPoeDF/EZSUZzCt+SPQGBbjD63nfapA0KWaOA7HY1Z8PzzuxF1Qjzjp60wjJNM5r/xPzYHf1HX2L2/C/YN1Mmm++71b1yLAk2knJfkzzXEvF2aPRbjFywNrQa1yt5aal90P7G4zviHAm8RLlDz/axqYSNsEBZfuE2gzgiwXUg4rLZ9xUG07AzlmlIzW) | MOL000296 | [hederagenin](http://tcmspw.com/molecule.php?qn=296) | 36.91 | 0.75 | 5.35 |
| [Rhizoma Curcumae](https://www.so.com/link?m=bxhhJwRTeWC/prtMCOClH8hPoeDF/EZSUZzCt+SPQGBbjD63nfapA0KWaOA7HY1Z8PzzuxF1Qjzjp60wjJNM5r/xPzYHf1HX2L2/C/YN1Mmm++71b1yLAk2knJfkzzXEvF2aPRbjFywNrQa1yt5aal90P7G4zviHAm8RLlDz/axqYSNsEBZfuE2gzgiwXUg4rLZ9xUG07AzlmlIzW) | MOL000906 | [wenjine](http://tcmspw.com/molecule.php?qn=906) | 47.93 | 0.27 | 5.1 |
| [Rhizoma Curcumae](https://www.so.com/link?m=bxhhJwRTeWC/prtMCOClH8hPoeDF/EZSUZzCt+SPQGBbjD63nfapA0KWaOA7HY1Z8PzzuxF1Qjzjp60wjJNM5r/xPzYHf1HX2L2/C/YN1Mmm++71b1yLAk2knJfkzzXEvF2aPRbjFywNrQa1yt5aal90P7G4zviHAm8RLlDz/axqYSNsEBZfuE2gzgiwXUg4rLZ9xUG07AzlmlIzW) | MOL000940 | [bisdemethoxycurcumin](http://tcmspw.com/molecule.php?qn=940) | 77.38 | 0.26 | 4.69 |
| Agrimonia pilosa | MOL000422 | kaempferol | 41.88 | 0.24 | 14.74 |
| Agrimonia pilosa | MOL000006 | luteolin | 36.16 | 0.25 | 15.94 |
| Agrimonia pilosa | MOL000098 | quercetin | 46.43 | 0.28 | 14.4 |
| Coptidis Rhizoma | MOL002897 | [epiberberine](http://tcmspw.com/molecule.php?qn=2897) | 43.09 | 0.78 | 6.1 |
| Coptidis Rhizoma | MOL002903 | [(R)-Canadine](http://tcmspw.com/molecule.php?qn=2903) | 55.37 | 0.77 | 6.41 |
| Coptidis Rhizoma | MOL002894 | [berberrubine](http://tcmspw.com/molecule.php?qn=2894) | 35.74 | 0.73 | 6.46 |
| Coptidis Rhizoma | MOL001454 | [berberine](http://tcmspw.com/molecule.php?qn=1454) | 36.86 | 0.78 | 6.57 |
| Coptidis Rhizoma | MOL002907 | [Corchoroside A_qt](http://tcmspw.com/molecule.php?qn=2907) | 104.95 | 0.78 | 6.68 |
| Coptidis Rhizoma | MOL002904 | [Berlambine](http://tcmspw.com/molecule.php?qn=2904) | 36.68 | 0.82 | 7.33 |
| Coptidis Rhizoma | MOL002668 | [Worenine](http://tcmspw.com/molecule.php?qn=2668) | 45.83 | 0.87 | 8.41 |
| Coptidis Rhizoma | MOL001458 | [coptisine](http://tcmspw.com/molecule.php?qn=1458) | 30.67 | 0.86 | 9.33 |
| Coptidis Rhizoma | MOL000762 | [Palmidin A](http://tcmspw.com/molecule.php?qn=762) | 35.36 | 0.65 | 33.17 |
| Coptidis Rhizoma | MOL000098 | [quercetin](http://tcmspw.com/molecule.php?qn=98) | 46.43 | 0.28 | 14.4 |
| Sophorae Flavescentis Radix | MOL001040 | [(2R)-5,7-dihydroxy-2-(4-hydroxyphenyl)chroman-4-one](http://tcmspw.com/molecule.php?qn=1040) | 42.36 | 0.21 | 16.83 |
| Sophorae Flavescentis Radix | MOL001484 | [Inermine](http://tcmspw.com/molecule.php?qn=1484) | 75.18 | 0.54 | 11.72 |
| Sophorae Flavescentis Radix | MOL003542 | [8-Isopentenyl-kaempferol](http://tcmspw.com/molecule.php?qn=3542) | 38.04 | 0.39 | 15.37 |
| Sophorae Flavescentis Radix | MOL003627 | [sophocarpine](http://tcmspw.com/molecule.php?qn=3627) | 64.26 | 0.25 | 5.54 |
| Sophorae Flavescentis Radix | MOL003648 | [Inermin](http://tcmspw.com/molecule.php?qn=3648) | 65.83 | 0.54 | 11.73 |
| Sophorae Flavescentis Radix | MOL003673 | [Wighteone](http://tcmspw.com/molecule.php?qn=3673) | 42.8 | 0.36 | 17.04 |
| Sophorae Flavescentis Radix | MOL003676 | [sophoramine](http://tcmspw.com/molecule.php?qn=3676) | 42.16 | 0.25 | 6.69 |
| Sophorae Flavescentis Radix | MOL003680 | [sophoridine](http://tcmspw.com/molecule.php?qn=3680) | 60.07 | 0.25 | 5.57 |
| Sophorae Flavescentis Radix | MOL000392 | [formononetin](http://tcmspw.com/molecule.php?qn=392) | 69.67 | 0.21 | 17.04 |
| Sophorae Flavescentis Radix | MOL004580 | [cis-Dihydroquercetin](http://tcmspw.com/molecule.php?qn=4580) | 66.44 | 0.27 | 14.51 |
| Sophorae Flavescentis Radix | MOL004941 | [(2R)-7-hydroxy-2-(4-hydroxyphenyl)chroman-4-one](http://tcmspw.com/molecule.php?qn=4941) | 71.12 | 0.18 | 18.09 |
| Sophorae Flavescentis Radix | MOL005100 | 5,7-dihydroxy-2-(3-hydroxy-4-me[thoxyphenyl)chroman-4-one](http://tcmspw.com/molecule.php?qn=5100) | 47.74 | 0.27 | 16.51 |
| Sophorae Flavescentis Radix | MOL005944 | [matrine](http://tcmspw.com/molecule.php?qn=5944) | 63.77 | 0.25 | 6.69 |
| Sophorae Flavescentis Radix | MOL006562 | (+)-7,11-dehydromatrine,(leontalb[inine)](http://tcmspw.com/molecule.php?qn=6562) | 62.08 | 0.25 | 6.46 |
| Sophorae Flavescentis Radix | MOL006563 | [(+)-9alpha-hydroxymatrine](http://tcmspw.com/molecule.php?qn=6563) | 32.04 | 0.29 | 4.55 |
| Sophorae Flavescentis Radix | MOL006564 | [(+)-allomatrine](http://tcmspw.com/molecule.php?qn=6564) | 58.87 | 0.25 | 5.49 |
| Sophorae Flavescentis Radix | MOL006565 | [AIDS211310](http://tcmspw.com/molecule.php?qn=6565) | 68.68 | 0.25 | 6.15 |
| Sophorae Flavescentis Radix | MOL006566 | [(+)-lehmannine](http://tcmspw.com/molecule.php?qn=6566) | 58.34 | 0.25 | 6.57 |
| Sophorae Flavescentis Radix | MOL006568 | [isosophocarpine](http://tcmspw.com/molecule.php?qn=6568) | 61.57 | 0.25 | 5.9 |
| Sophorae Flavescentis Radix | MOL006570 | [(-)-9alpha-hydroxysophoramine](http://tcmspw.com/molecule.php?qn=6570) | 35.23 | 0.29 | 4.28 |
| Sophorae Flavescentis Radix | MOL006571 | [anagyrine](http://tcmspw.com/molecule.php?qn=6571) | 62.01 | 0.24 | 4.74 |
| Sophorae Flavescentis Radix | MOL006572 | [1,4-diazaindan-type,alkaloid,flavascensine](http://tcmspw.com/molecule.php?qn=6572) | 34.64 | 0.24 | 5.62 |
| Sophorae Flavescentis Radix | MOL006573 | [13,14-dehydrosophoridine](http://tcmspw.com/molecule.php?qn=6573) | 65.34 | 0.25 | 6.24 |
| Sophorae Flavescentis Radix | MOL006582 | [5α,9α-dihydroxymatrine](http://tcmspw.com/molecule.php?qn=6582) | 40.93 | 0.32 | 4.59 |
| Sophorae Flavescentis Radix | MOL006583 | [7,11-dehydromatrine](http://tcmspw.com/molecule.php?qn=6583) | 44.43 | 0.25 | 5.22 |
| Sophorae Flavescentis Radix | MOL006596 | [Glyceollin](http://tcmspw.com/molecule.php?qn=6596) | 97.27 | 0.76 | 6.24 |
| Sophorae Flavescentis Radix | MOL006604 | [(2S)-7-hydroxy-2-(4-hydroxyphenyl)-5-methoxy-8-(3-methylbut-2-enyl)chroman-4-one](http://tcmspw.com/molecule.php?qn=6604) | 48.09 | 0.39 | 15.54 |
| Sophorae Flavescentis Radix | MOL006613 | [kushenin](http://tcmspw.com/molecule.php?qn=6613) | 47.62 | 0.38 | 8.86 |
| Sophorae Flavescentis Radix | MOL006619 | [kushenol J](http://tcmspw.com/molecule.php?qn=6619) | 51.39 | 0.74 | 13.81 |
| Sophorae Flavescentis Radix | MOL006620 | [kushenol J_qt](http://tcmspw.com/molecule.php?qn=6620) | 50.86 | 0.24 | 15.78 |
| Sophorae Flavescentis Radix | MOL006622 | [kushenol O](http://tcmspw.com/molecule.php?qn=6622) | 42.41 | 0.76 | 15.73 |
| Sophorae Flavescentis Radix | MOL006632 | [kushenol,t](http://tcmspw.com/molecule.php?qn=6623) | 51.28 | 0.64 | 17.47 |
| Sophorae Flavescentis Radix | MOL006626 | [leachianone,g](http://tcmspw.com/molecule.php?qn=6626) | 60.97 | 0.4 | 15.53 |
| Sophorae Flavescentis Radix | MOL006627 | [Lehmanine](http://tcmspw.com/molecule.php?qn=6627) | 62.23 | 0.25 | 6 |
| Sophorae Flavescentis Radix | MOL006628 | [(+)-Lupanine](http://tcmspw.com/molecule.php?qn=6628) | 52.71 | 0.24 | 4.25 |
| Sophorae Flavescentis Radix | MOL006630 | [Norartocarpetin](http://tcmspw.com/molecule.php?qn=6630) | 54.93 | 0.24 | 17.23 |
| Sophorae Flavescentis Radix | MOL000456 | [Phaseolin](http://tcmspw.com/molecule.php?qn=456) | 78.2 | 0.73 | 7.56 |
| Sophorae Flavescentis Radix | MOL006649 | [sophranol](http://tcmspw.com/molecule.php?qn=6649) | 55.42 | 0.28 | 4.94 |
| Sophorae Flavescentis Radix | MOL006650 | [(-)-Maackiain-3-O-glucosyl-6'-O-malonate](http://tcmspw.com/molecule.php?qn=6650) | 48.69 | 0.52 | 20.42 |
| Sophorae Flavescentis Radix | MOL006652 | [trifolrhizin](http://tcmspw.com/molecule.php?qn=6652) | 48.53 | 0.74 | 18.51 |
| Sophorae Flavescentis Radix | MOL000006 | [luteolin](http://tcmspw.com/molecule.php?qn=98) | 36.16 | 0.25 | 15.94 |
| Sophorae Flavescentis Radix | MOL000098 | quercetin | 46.43 | 0.28 | 14.4 |
| Coicis Semen | MOL001323 | [Sitosterol alpha1](http://tcmspw.com/molecule.php?qn=1323) | 43.28 | 0.78 | 5.64 |
| Coicis Semen | MOL001494 | [Mandenol](http://tcmspw.com/molecule.php?qn=1494) | 42 | 0.19 | 5.39 |
| Coicis Semen | MOL002882 | [[(2R)-2,3-dihydroxypropyl] (Z)-octadec-9-enoate](http://tcmspw.com/molecule.php?qn=2882) | 34.13 | 0.3 | 5.19 |
| Coicis Semen | MOL000359 | [sitosterol](http://tcmspw.com/molecule.php?qn=359) | 36.91 | 0.75 | 5.37 |
| Coicis Semen | MOL000449 | [Stigmasterol](http://tcmspw.com/molecule.php?qn=449) | 43.83 | 0.76 | 5.57 |
| Coicis Semen | MOL008121 | [2-Monoolein](http://tcmspw.com/molecule.php?qn=8121) | 34.23 | 0.29 | 4.41 |
| Coicis Semen | MOL000953 | [CLR](http://tcmspw.com/molecule.php?qn=953) | 37.87 | 0.68 | 4.52 |

**Supplemental Table S2**. WJR potential therapeutic targets and CRC-related targets

| **WJR therapeutic targets** | **CRC-related targets** |
| --- | --- |
| MMP2 | MMP2 |
| CD44 | CD44 |
| HSPB1 | HSPB1 |
| PLAU | PLAU |
| PTEN | PTEN |
| NOS2 | NOS2 |
| CCNB1 | CCNB1 |
| MET | MET |
| IRF1 | IRF1 |
| ALOX5 | ALOX5 |
| GJA1 | GJA1 |
| CLDN4 | CLDN4 |
| BIRC5 | BIRC5 |
| CDK1 | CDK1 |
| CHEK1 | CHEK1 |
| BCL2 | BCL2 |
| CYP1A1 | CYP1A1 |
| E2F1 | E2F1 |
| CASP8 | CASP8 |
| PPARG | PPARG |
| CRP | CRP |
| GSTP1 | GSTP1 |
| CXCL8 | CXCL8 |
| SELE | SELE |
| AHR | AHR |
| NFE2L2 | NFE2L2 |
| MAPK14 | MAPK14 |
| TNF | TNF |
| RAF1 | RAF1 |
| EGF | EGF |
| IL1A | IL1A |
| MPO | MPO |
| PCNA | PCNA |
| SPP1 | SPP1 |
| F2 | F2 |
| PTGS2 | PTGS2 |
| ADH1C | ADH1C |
| RUNX1T1 | RUNX1T1 |
| CCND1 | CCND1 |
| ESR1 | ESR1 |
| CDK4 | CDK4 |
| CASP7 | CASP7 |
| CTSD | CTSD |
| VEGFA | VEGFA |
| TGFB1 | TGFB1 |
| MYC | MYC |
| CCNA2 | CCNA2 |
| GSK3B | GSK3B |
| MCL1 | MCL1 |
| CCL2 | CCL2 |
| MMP1 | MMP1 |
| STAT1 | STAT1 |
| IL6 | IL6 |
| CASP3 | CASP3 |
| HSP90AA1 | HSP90AA1 |
| PPARD | PPARD |
| KDR | KDR |
| ABCG2 | ABCG2 |
| HMOX1 | HMOX1 |
| MMP3 | MMP3 |
| CYP1A2 | CYP1A2 |
| GSTM1 | GSTM1 |
| IL10 | IL10 |
| MAPK1 | MAPK1 |
| IGF2 | IGF2 |
| PIK3CG | PIK3CG |
| MDM2 | MDM2 |
| EGFR | EGFR |
| NQO1 | NQO1 |
| SOD1 | SOD1 |
| IL2 | IL2 |
| ERBB3 | ERBB3 |
| ERBB2 | ERBB2 |
| IFNG | IFNG |
| FOS | FOS |
| HSPA5 | HSPA5 |
| IL4 | IL4 |
| TOP2A | TOP2A |
| MAPK8 | MAPK8 |
| ICAM1 | ICAM1 |
| CAV1 | CAV1 |
| BCL2L1 | BCL2L1 |
| CHEK2 | CHEK2 |
| RELA | RELA |
| HIF1A | HIF1A |
| NOS3 | NOS3 |
| RB1 | RB1 |
| CDK2 | CDK2 |
| RUNX2 | RUNX2 |
| PGR | PGR |
| ODC1 | ODC1 |
| E2F2 | E2F2 |
| TYR | TYR |
| SERPINE1 | SERPINE1 |
| VCAM1 | VCAM1 |
| TP53 | TP53 |
| INSR | INSR |
| CASP9 | CASP9 |
| ADH1B | ADH1B |
| CDKN1A | CDKN1A |
| AKT1 | AKT1 |
| PRKACA | PRKACA |
| SIRT1 | SIRT1 |
| IL1B | IL1B |
| AKR1C3 | AKR1C3 |
| NFKBIA | NFKBIA |
| IGFBP3 | IGFBP3 |
| PTGS1 | PTGS1 |
| JUN | JUN |
| AR | AR |
| ESR2 | ESR2 |
| BAX | BAX |
| PRKCA | PRKCA |
| CD40LG | CD40LG |
| RASSF1 | RASSF1 |
| CYP3A4 | CYP3A4 |
| CYP1B1 | CYP1B1 |
| PRSS1 | PRSS1 |
| MMP9 | MMP9 |
| TOP1 | TOP1 |
| PTGER3 | ERBB4 |
| KCNMA1 | ERCC5 |
| SLC6A2 | ITGA5 |
| XDH | SPINK1 |
| ADRA2C | MIR193B |
| EIF6 | EDN1 |
| DCAF5 | TKT |
| SLC6A4 | NBN |
| CHRM5 | MIR31 |
| PCOLCE | SNHG16 |
| PRKCB | CXCR4 |
| RXRA | BID |
| ACACA | COX5A |
| COL1A1 | FAN1 |
| PPP3CA | AIFM1 |
| HTR2C | IGF1 |
| DPP4 | MIR146A |
| DRD5 | SOS1 |
| MAOA | TGFB2 |
| PON1 | MIR20A |
| PSMD3 | GAS5 |
| ADRA2A | BCAR4 |
| F7 | MIRLET7G |
| KCNH2 | RAC1 |
| DUOX2 | POLK |
| HTR3A | PAK1 |
| PTGES | NTRK1 |
| CHRM1 | MIR328 |
| CXCL11 | GDF15 |
| ADRA1A | ANXA1 |
| CHRNA2 | CYCS |
| PIM1 | TPX2 |
| NR1I3 | EP300 |
| THBD | AXIN1 |
| NR3C1 | MMP7 |
| CHRM4 | SETD2 |
| CAMKK1 | MIR451A |
| ADRB1 | MMP13 |
| ADRA1B | SERPINB5 |
| HSD3B1 | SEC23B |
| CHUK | LGR6 |
| OLR1 | ZEB1 |
| SLPI | ERG |
| NCF1 | CRCS11 |
| HAS2 | HLTF |
| TNKS | FAT4 |
| GABRA2 | SOD2 |
| PDE3A | MIR15A |
| ADRB2 | RPL34-AS1 |
| POR | MSH3 |
| ADRA1D | CD4 |
| CAMKKA | FGFR1 |
| GABRA6 | BAK1 |
| MT-ND6 | CEACAM6 |
| NUF2 | TUG1 |
| ACHE | SDHB |
| COL8A1 | BBC3 |
| GABRA1 | MAX |
| NR3C2 | SOX4 |
| MGAM | AFAP1-AS1 |
| SULT1E1 | SLCO1B3 |
| PPARA | UGT1A1 |
| MAOB | MIR200B |
| BIRC4 | NRP1 |
| ELK1 | NANOG |
| AHSA1 | CDH13 |
| NPEPPS | LEP |
| PLAT | HEIH |
| CXCL2 | FASN |
| IKBKB | PHLPP2 |
| NKX3-1 | CHSY1 |
| HSD3B2 | SHC1 |
| HK2 | BRCA1 |
| OPRD1 | CYP2D6 |
| F10 | PTPN12 |
| AKR1B1 | FAM83H-AS1 |
| HSP90AB1 | RUNX3 |
| NCOA1 | TGM2 |
| IGHG1 | MIR129-2 |
| SLC2A4 | MIR30A |
| MAP2 | BMPR1A |
| HSF1 | MIR132 |
| PDE10A | PIK3R5 |
| ACP3 | TIMP3 |
| CHRM2 | LINC00460 |
| gyrB | EPHA2 |
| DPEP1 | TLR1 |
| OPRM1 | MIR18A |
| PKIA | TEK |
| CXCL10 | SQSTM1 |
| HSP90A | MIR149 |
| IER3IP1 | NFATC1 |
| ATP5F1B | HNF1A-AS1 |
| DIO1 | MTHFR |
| RASA1 | PTPN11 |
| LYZ | SMAD3 |
| NCOA2 | PDCD4 |
| GABRA5 | ATF3 |
| ADCY2 | CCN4 |
| CHRM3 | FOXP3 |
| HPSE | GNAS |
| F3 | PLK1 |
| HTR2A | DDB2 |
| NR1I2 | WNT8B |
| PYGM | SLC16A7 |
| Ptpn1 | LINC00261 |
| GSTM2 | ITGB4 |
| CTRB1 | MT-ND4L |
| APP A4 | MIR148A |
| GABRA3 | RAD23B |
| SCN5A | MIR204 |
| GRIA2 | DICER1 |
| SLC6A3 | ZNF148 |
| LTA4H | MIR19A |
| CHRNA7 | TNFRSF9 |
| RXRB | H2AC18 |
|  | CCNE1 |
|  | IFNA2 |
|  | PLA2G2A |
|  | MUC3A |
|  | FOLH1 |
|  | FOXM1 |
|  | LGALS1 |
|  | ALK |
|  | CASP10 |
|  | EZH2 |
|  | APOE |
|  | MSH6 |
|  | MUC16 |
|  | ALDH2 |
|  | FAS |
|  | MIR127 |
|  | CASC8 |
|  | SRSF6 |
|  | GRB2 |
|  | BDNF |
|  | LCOR |
|  | HBEGF |
|  | MIR378A |
|  | CDKN1B |
|  | MIR99A |
|  | NTHL1 |
|  | PTK2 |
|  | MIR128-2 |
|  | MIR192 |
|  | MIR34B |
|  | SHBG |
|  | MIR454 |
|  | MIR223 |
|  | ABCA1 |
|  | CSF1 |
|  | MIR200C |
|  | TNFSF10 |
|  | SMARCB1 |
|  | CD82 |
|  | MIR193A |
|  | SETDB1 |
|  | MUC1 |
|  | SKP2 |
|  | TNFRSF10A |
|  | GHET1 |
|  | PTHLH |
|  | NPTN-IT1 |
|  | MIR27A |
|  | CD36 |
|  | MUC7 |
|  | PIK3CB |
|  | MIR10A |
|  | MIR215 |
|  | MIR196B |
|  | IDH2 |
|  | MIR320A |
|  | XRCC6 |
|  | NPM1 |
|  | DNMT3B |
|  | GUCY2C |
|  | DCLK1 |
|  | TLR6 |
|  | ELAC2 |
|  | ASS1 |
|  | TERC |
|  | EDNRA |
|  | CDKN2B-AS1 |
|  | MIR9-1 |
|  | PTENP1 |
|  | CXCR2 |
|  | IRS1 |
|  | MIRLET7C |
|  | ESRRA |
|  | ATR |
|  | CD40 |
|  | MAPRE1 |
|  | FGF8 |
|  | DPYD |
|  | APOB |
|  | CDC25C |
|  | TSC2 |
|  | MIR23A |
|  | IGFBP7 |
|  | CDH2 |
|  | QKI |
|  | EZR |
|  | WNT3 |
|  | NCAM1 |
|  | CCAT2 |
|  | ATF1 |
|  | FGFR3 |
|  | MIR203A |
|  | DPP10-AS1 |
|  | KRAS |
|  | MIR137 |
|  | CDX2 |
|  | FGF1 |
|  | HOXB13 |
|  | IL6R |
|  | MNX1-AS1 |
|  | FUT4 |
|  | PPP2R1B |
|  | JAG1 |
|  | CHGA |
|  | ALOX15 |
|  | HSPA4 |
|  | RHOA |
|  | NEAT1 |
|  | MUTYH |
|  | FANCC |
|  | HSPA8 |
|  | RARB |
|  | TP73 |
|  | PRKCD |
|  | MIR372 |
|  | MIR92A1 |
|  | TPBG |
|  | RET |
|  | XPC |
|  | TCF4 |
|  | HLA-DQB1 |
|  | MT-CYB |
|  | NRAS |
|  | AURKA |
|  | MAPK9 |
|  | MIR574 |
|  | SRD5A2 |
|  | MIR122 |
|  | EGR1 |
|  | CRCS6 |
|  | PAEP |
|  | COLCA1 |
|  | OGG1 |
|  | HDAC9 |
|  | MIR206 |
|  | TIAM1 |
|  | AFP |
|  | MIR182 |
|  | LNCRNA-ATB |
|  | FN1 |
|  | MTR |
|  | ABCB1 |
|  | DHFR |
|  | DLEC1 |
|  | MIR144 |
|  | LLGL1 |
|  | KLF6 |
|  | PIK3R3 |
|  | MIR181A2 |
|  | KLF4 |
|  | ACVR2A |
|  | IFI27 |
|  | IL17A |
|  | CLCA1 |
|  | WNT3A |
|  | LEPR |
|  | MTOR |
|  | MIR29B1 |
|  | SFRP2 |
|  | BMP6 |
|  | MIR124-1 |
|  | CYP2A6 |
|  | PCAT1 |
|  | CASP5 |
|  | STUB1 |
|  | KDM1A |
|  | WT1 |
|  | ILK |
|  | BNIP3 |
|  | SNAI1 |
|  | NCOA3 |
|  | LEF1 |
|  | CYP19A1 |
|  | KCNQ1OT1 |
|  | CCAT1 |
|  | SERPINB2 |
|  | SOX9 |
|  | MIR424 |
|  | SATB2 |
|  | CDK6 |
|  | RNY1 |
|  | PDGFRB |
|  | CSK |
|  | MUC5B |
|  | CTSB |
|  | KLLN |
|  | NCRUPAR |
|  | TH |
|  | MUC12 |
|  | FZD7 |
|  | UMPS |
|  | PXN |
|  | LCK |
|  | MIR29B2 |
|  | MUC2 |
|  | FURIN |
|  | CASC19 |
|  | WNT6 |
|  | GNRH1 |
|  | PDGFD |
|  | ERCC1 |
|  | SULT1A1 |
|  | BLM |
|  | AGO2 |
|  | CREBBP |
|  | ITGB1 |
|  | SMAD7 |
|  | MIR7-3 |
|  | VEGFC |
|  | MAP2K2 |
|  | ABCC2 |
|  | PDGFB |
|  | MIR32 |
|  | TFE3 |
|  | SELP |
|  | CALR |
|  | VIM |
|  | TLR8 |
|  | ZEB1-AS1 |
|  | LGALS3BP |
|  | CSMD3 |
|  | FGFR2 |
|  | ABCC3 |
|  | ITGAV |
|  | XRCC3 |
|  | CRCS7 |
|  | CSF3 |
|  | KIT |
|  | PRKAR1A |
|  | HLA-A |
|  | ATG5 |
|  | KRT8 |
|  | SHH |
|  | UCA1 |
|  | MIR210 |
|  | FHIT |
|  | FER1L4 |
|  | MIR106B |
|  | JAK2 |
|  | LGR5 |
|  | ANTXR1 |
|  | NR1H2 |
|  | PDPK1 |
|  | TNFRSF1A |
|  | ETV4 |
|  | STAT3 |
|  | RAD54B |
|  | CD8A |
|  | MIR542 |
|  | MBD4 |
|  | GLI1 |
|  | BARD1 |
|  | CTBP1 |
|  | MAD1L1 |
|  | XIAP |
|  | CA9 |
|  | MIR103A1 |
|  | EIF2B2 |
|  | WNT5A |
|  | ENO2 |
|  | LGALS4 |
|  | SDHD |
|  | EIF5A2 |
|  | B2M |
|  | NF1 |
|  | LRP5 |
|  | CLMAT3 |
|  | TMEM238L |
|  | RAD51C |
|  | KLK4 |
|  | BCAR1 |
|  | CTNNB1 |
|  | MIR221 |
|  | H19 |
|  | MIR183 |
|  | MIR141 |
|  | TLR5 |
|  | TGFBR1 |
|  | ZNF217 |
|  | FCGR3A |
|  | CYP2C9 |
|  | DNMT3A |
|  | CDC42 |
|  | VWF |
|  | BAD |
|  | TBX1 |
|  | MIR24-1 |
|  | GALNT12 |
|  | ITGA6 |
|  | SEPTIN9 |
|  | CYP2E1 |
|  | IGFBP2 |
|  | NOTCH2 |
|  | MSR1 |
|  | LAMC1-AS1 |
|  | E2F4 |
|  | S100B |
|  | BMP2 |
|  | TUSC7 |
|  | ATM |
|  | TP53COR1 |
|  | SST |
|  | LIG4 |
|  | EREG |
|  | BMP4 |
|  | WIF1 |
|  | DVL1 |
|  | PRMT1 |
|  | CALB2 |
|  | ABCB5 |
|  | TYMS |
|  | IRS2 |
|  | ZFAS1 |
|  | MELK |
|  | GPX2 |
|  | TJP1 |
|  | CSE1L |
|  | HRAS |
|  | CD46 |
|  | PTPRT |
|  | RALGDS |
|  | ICOSLG |
|  | RNF43 |
|  | HOTAIRM1 |
|  | CCKBR |
|  | MAP2K4 |
|  | ACSL5 |
|  | UGT1A6 |
|  | RNY3 |
|  | U2AF1 |
|  | KAT5 |
|  | CDKN2B |
|  | SMAD4 |
|  | CASC2 |
|  | MAP3K6 |
|  | PROS1 |
|  | FGFR4 |
|  | HPGD |
|  | XRCC1 |
|  | STAT6 |
|  | IL3 |
|  | AREG |
|  | GLI3 |
|  | ITGA2 |
|  | PTCH1 |
|  | MME |
|  | S100A6 |
|  | APEX1 |
|  | AMACR |
|  | GAST |
|  | PTPA |
|  | GREM1 |
|  | NTS |
|  | FZD10 |
|  | FEZF1-AS1 |
|  | NTRK3 |
|  | PLK4 |
|  | KEAP1 |
|  | TMEFF2 |
|  | MUC4 |
|  | WWOX |
|  | CRCS8 |
|  | TIMP1 |
|  | MGMT |
|  | BUB1 |
|  | NKX2-1 |
|  | YES1 |
|  | ALDOA |
|  | IL13 |
|  | MIR21 |
|  | LASP1 |
|  | FOXO1 |
|  | PANDAR |
|  | MIR125A |
|  | CCND3 |
|  | PTTG1 |
|  | TLR3 |
|  | ANXA5 |
|  | WNT16 |
|  | MMP12 |
|  | STMN1 |
|  | GUCA2A |
|  | SLC2A1 |
|  | DUXAP10 |
|  | CRNDE |
|  | PTGER4 |
|  | EPCAM |
|  | MIR15B |
|  | HMGA2 |
|  | EPOR |
|  | EIF4G1 |
|  | MIR185 |
|  | RHO |
|  | MIR338 |
|  | BSG |
|  | ANGPT2 |
|  | MIRLET7A1 |
|  | MIR335 |
|  | NNT-AS1 |
|  | PHLPP1 |
|  | SYP |
|  | PARP1 |
|  | MMP11 |
|  | DNAJC6 |
|  | VDR |
|  | ACE |
|  | YBX1 |
|  | ANXA2 |
|  | CDH3 |
|  | PRKCE |
|  | FH |
|  | PDPN |
|  | MIR423 |
|  | FASLG |
|  | ENG |
|  | MIR126 |
|  | SPRY4-IT1 |
|  | GGT1 |
|  | MIR129-1 |
|  | WNT1 |
|  | KRT14 |
|  | TCF7L1 |
|  | PDGFRL |
|  | PRKN |
|  | EPHA3 |
|  | MIR195 |
|  | INS |
|  | FLT1 |
|  | MEN1 |
|  | SNHG20 |
|  | ALOX12 |
|  | RARA |
|  | COLCA2 |
|  | HOTAIR |
|  | EIF4E |
|  | PECAM1 |
|  | MIR205 |
|  | CRCS2 |
|  | GUCA2B |
|  | SMAD2 |
|  | HOXA11-AS |
|  | EBAG9 |
|  | UROD |
|  | PRKCZ |
|  | MIR145 |
|  | LRP6 |
|  | AXL |
|  | CCR6 |
|  | GATA3 |
|  | MIR135A1 |
|  | BRCA2 |
|  | CD24 |
|  | TTR |
|  | FLCN |
|  | NAMPT |
|  | PTGER2 |
|  | ERCC4 |
|  | TG |
|  | MIR34A |
|  | BCL2L11 |
|  | TGFB3 |
|  | CTNNA1 |
|  | MIR497 |
|  | MIR222 |
|  | NTRK2 |
|  | S100A4 |
|  | MAP2K5 |
|  | CRCS5 |
|  | BLACAT1 |
|  | TLR10 |
|  | CD80 |
|  | GADD45A |
|  | MIR214 |
|  | TSC1 |
|  | PSMB7 |
|  | FPGS |
|  | DNMT1 |
|  | LRP1 |
|  | SOX5 |
|  | MIR486-1 |
|  | BRIP1 |
|  | MIR532 |
|  | PSG2 |
|  | REG4 |
|  | PALB2 |
|  | MMP14 |
|  | DANCR |
|  | CLDN3 |
|  | AKT2 |
|  | SNHG5 |
|  | LIFR |
|  | MUC6 |
|  | TFDP1 |
|  | SP1 |
|  | AXIN2 |
|  | DMBT1 |
|  | BACE1-AS |
|  | MIR23B |
|  | AFAP1 |
|  | MIR200A |
|  | FSCN1 |
|  | ZFP36L1 |
|  | PIK3CA |
|  | FLT3 |
|  | MIRLET7B |
|  | CTLA4 |
|  | ADORA1 |
|  | REEP5 |
|  | KDM4C |
|  | WNT2 |
|  | FADD |
|  | CREB1 |
|  | MIR675 |
|  | TLR7 |
|  | IGFBP1 |
|  | MIR196A1 |
|  | DDIT3 |
|  | CEACAM5 |
|  | LGALS3 |
|  | UGT1A7 |
|  | CDH1 |
|  | FUT3 |
|  | SMARCA4 |
|  | DIABLO |
|  | NAT2 |
|  | RALA |
|  | MIR135B |
|  | MUCL1 |
|  | XPA |
|  | LINC00858 |
|  | MIR142 |
|  | KRT20 |
|  | AKAP12 |
|  | ENPP7 |
|  | FGF2 |
|  | CLU |
|  | STAT5B |
|  | XIST |
|  | CXCL12 |
|  | PPP2R1A |
|  | MIR93 |
|  | NTN1 |
|  | MIR224 |
|  | WNT10A |
|  | RRM2 |
|  | MRE11 |
|  | KLF5 |
|  | EPHX1 |
|  | SIL1 |
|  | PRTN3 |
|  | IGF1R |
|  | WNT11 |
|  | DUXAP9 |
|  | PRKD1 |
|  | MIR199B |
|  | RECK |
|  | MIR191 |
|  | THBS1 |
|  | ALDH1A1 |
|  | MTAP |
|  | MIR339 |
|  | ADORA3 |
|  | HPRT1 |
|  | MIRLET7I |
|  | RNASEL |
|  | H2AX |
|  | TNS4 |
|  | PIK3R2 |
|  | ABL1 |
|  | PTPRG |
|  | MAPK3 |
|  | MIR143 |
|  | PRDM10 |
|  | PSMA7 |
|  | MAP2K7 |
|  | GZMB |
|  | VIP |
|  | RAD54L |
|  | CYTOR |
|  | HDAC2 |
|  | HLA-G |
|  | GPER1 |
|  | MTDH |
|  | WNT2B |
|  | ETS1 |
|  | PMAIP1 |
|  | RAD50 |
|  | MIR34C |
|  | MYCN |
|  | SYNE1 |
|  | MIR181A1 |
|  | EIF4EBP1 |
|  | CXCL1 |
|  | MIR100 |
|  | GUSB |
|  | HNF4A |
|  | TNFRSF10B |
|  | TERT |
|  | PAX8 |
|  | MIR106A |
|  | KRT18 |
|  | MIRLET7F1 |
|  | MACC1 |
|  | GRN |
|  | GRPR |
|  | MIR133B |
|  | SOCS1 |
|  | MIR155 |
|  | FZD8 |
|  | MCC |
|  | TGFA |
|  | ANPEP |
|  | PROM1 |
|  | TUSC8 |
|  | MIR101-1 |
|  | MIR10B |
|  | PRLR |
|  | TRIM28 |
|  | SOX2-OT |
|  | MIR96 |
|  | CEACAM7 |
|  | WRN |
|  | BAP1 |
|  | MIR25 |
|  | MSH2 |
|  | SLC29A1 |
|  | ZEB2 |
|  | SRC |
|  | FBXW7 |
|  | MTA1 |
|  | CASR |
|  | MIR30E |
|  | RAD51L3-RFFL |
|  | NR0B2 |
|  | TFAP2A |
|  | MIR16-1 |
|  | BUB1B |
|  | MT-CO1 |
|  | DKK1 |
|  | IL1RN |
|  | MAGEA1 |
|  | ERCC6 |
|  | KRT7 |
|  | TDGF1 |
|  | MIR296 |
|  | MIR139 |
|  | CADM1 |
|  | CDKN1C |
|  | POLE |
|  | PTGES2 |
|  | GFAP |
|  | CYP17A1 |
|  | MIR22 |
|  | MIR29C |
|  | TLR4 |
|  | KRT19 |
|  | SDHC |
|  | ADAMTS13 |
|  | PLG |
|  | MIRLET7D |
|  | TCF3 |
|  | TP53TG1 |
|  | HFE |
|  | SUFU |
|  | MIR324 |
|  | MKI67 |
|  | STS |
|  | MIR17 |
|  | MIR107 |
|  | LINC01133 |
|  | CCL5 |
|  | MLH3 |
|  | ACTG2 |
|  | LINC-ROR |
|  | MIR373 |
|  | JUP |
|  | MIR140 |
|  | STK11 |
|  | TGFBR2 |
|  | TLR9 |
|  | DEAF1 |
|  | LOX |
|  | MIR455 |
|  | HOXA-AS2 |
|  | IFNA1 |
|  | HNF1B |
|  | CCN1 |
|  | CTAG1B |
|  | HLA-DRB1 |
|  | PDCD1 |
|  | MEG3 |
|  | FGF3 |
|  | RAD51 |
|  | RAD51D |
|  | MIR146B |
|  | CAT |
|  | HLA-B |
|  | TCF7L2 |
|  | PKM |
|  | LAMC2 |
|  | JAK1 |
|  | CTNND1 |
|  | MT-CO2 |
|  | ELAVL1 |
|  | GCNT3 |
|  | EXO1 |
|  | CCND2 |
|  | SNHG12 |
|  | CFLAR |
|  | PIK3R1 |
|  | GPC3 |
|  | GRP |
|  | CDKN2C |
|  | PDGFRA |
|  | PTPRJ |
|  | SNHG6 |
|  | MIR130B |
|  | MIR150 |
|  | EPHB6 |
|  | DLC1 |
|  | MIR199A1 |
|  | VEGFD |
|  | ZFHX3 |
|  | FZD3 |
|  | IDH1 |
|  | CDCP1 |
|  | FZD6 |
|  | SEMA4A |
|  | SNHG1 |
|  | XRCC5 |
|  | PAX3 |
|  | POU5F1 |
|  | HDAC1 |
|  | YAP1 |
|  | MUC5AC |
|  | MIR342 |
|  | BMI1 |
|  | MALAT1 |
|  | CLDN1 |
|  | MAP2K1 |
|  | NFKB1 |
|  | ABCC1 |
|  | MIR27B |
|  | TIMP2 |
|  | ITGB3 |
|  | MIR345 |
|  | MIR422A |
|  | CPE |
|  | WRAP53 |
|  | MORC2 |
|  | IGF2R |
|  | PTPRU |
|  | POLD1 |
|  | MIR196A2 |
|  | ST6GAL1 |
|  | PMS1 |
|  | AGBL5 |
|  | CBR3-AS1 |
|  | TCF7 |
|  | KITLG |
|  | TXN |
|  | SPARC |
|  | DDR2 |
|  | FZD1 |
|  | BECN1 |
|  | ALB |
|  | CEACAM1 |
|  | DCC |
|  | NOD2 |
|  | MIR24-2 |
|  | PRNCR1 |
|  | MLH1 |
|  | MIR95 |
|  | PHB |
|  | HLA-C |
|  | WNT5B |
|  | ARAF |
|  | FOXO3 |
|  | NORAD |
|  | GAPDH |
|  | APC |
|  | HULC |
|  | TLR2 |
|  | UHRF1 |
|  | GPA33 |
|  | PVT1 |
|  | P2RX7 |
|  | FZD4 |
|  | ING1 |
|  | CDKN2A |
|  | ETV6 |
|  | TMSB4X |
|  | PSCA |
|  | RECQL4 |
|  | CEBPB |
|  | BCL10 |
|  | CASP2 |
|  | HMGA1 |
|  | SERPINA3 |
|  | BCYRN1 |
|  | CSF1R |
|  | USP28 |
|  | NAT1 |
|  | NOTCH3 |
|  | MIR186 |
|  | NF2 |
|  | NRG1 |
|  | TFF1 |
|  | BANCR |
|  | EPHB2 |
|  | SNAI2 |
|  | PLAUR |
|  | FLT4 |
|  | CDKN3 |
|  | NME1 |
|  | GAPLINC |
|  | MIR625 |
|  | MIR483 |
|  | SCG5 |
|  | E2F3 |
|  | HDC |
|  | TFF3 |
|  | BRAF |
|  | TWIST1 |
|  | FLNA |
|  | APRT |
|  | CRCS9 |
|  | NOTCH1 |
|  | CD274 |
|  | PTPN13 |
|  | SFRP1 |
|  | AKT3 |
|  | DAPK1 |
|  | KLK3 |
|  | CYP24A1 |
|  | CASC11 |
|  | FOSL1 |
|  | GSTT1 |
|  | TCOF1 |
|  | KMT2C |
|  | LINC00472 |
|  | EVL |
|  | HGF |
|  | EPHB4 |
|  | TYMP |
|  | HIF1A-AS1 |
|  | MIR29A |
|  | ARID1A |
|  | ACSL4 |
|  | IL7 |
|  | PPM1D |
|  | SOX2 |
|  | HOTTIP |
|  | TP63 |
|  | SDHA |
|  | VHL |
|  | COMT |
|  | CACNA1G |
|  | SAT1 |
|  | MIR26A1 |
|  | ROS1 |
|  | DACT1 |
|  | APC2 |
|  | MDM4 |
|  | ERCC2 |
|  | CEACAM3 |
|  | CDX1 |
|  | MIR375 |
|  | AGR2 |
|  | FTX |
|  | FANCG |
|  | TINCR |
|  | FANCD2 |
|  | HMGB1 |
|  | CSF2 |
|  | PMS2 |
|  | BIRC3 |
|  | RPS6KB1 |
|  | XRCC2 |
|  | ADIPOQ |

**Figures and figure legends**

**Supplementary Figure 1.** Cell viability was determined by MTT assay in 293T cell. NC, control group; LD, low-dose group of WJR; MD, middle-dose group of WJR; HD, high-dose group of WJR; PC, positive group; WJR, Wenzi Jiedu Recipe.

**
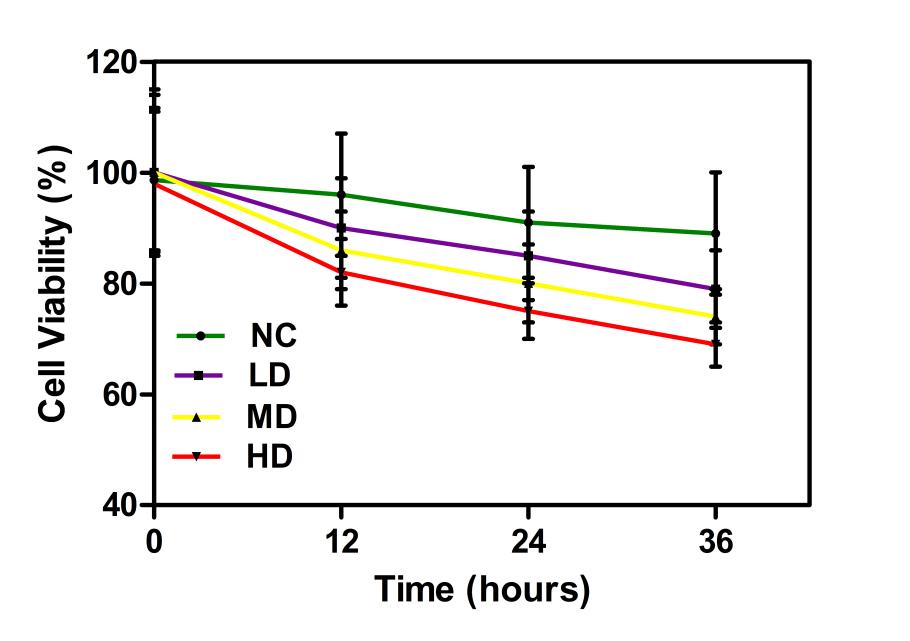
**
